# Mercury distribution in plants and soils from the former mining area of Abbadia San Salvatore (Tuscany, Central Italy)

**DOI:** 10.1007/s10653-023-01739-w

**Published:** 2023-08-30

**Authors:** Federica Meloni, Alessandro Farieri, Pablo L. Higueras, José M. Esbrí, Barbara Nisi, Jacopo Cabassi, Daniele Rappuoli, Orlando Vaselli

**Affiliations:** 1 Department of Earth Sciences, Via G. La Pira, 4-50121 Florence, Italy; 2 CNR-IGG Institute of Geosciences and Earth Resources, Via G. La Pira, 4-50121 Florence, Italy; 3 Instituto de Geología Aplicada, EIMIA - Pl. Manuel Meca 1 13400 Almadén, Ciudad Real, Spain; 4 Departament of Mineralogy and Petrology, (UCM), C. de José Antonio Novais, 12, 28040 Madrid, Spain; 5 Unione Dei Comuni Amiata Val d’Orcia, Unità Di Bonifica, Via Grossetana, 209-53025 Piancastagnaio, Siena, Italy; 6 Parco Museo Minerario Di Abbadia San Salvatore - Via Suor Gemma, 53021 Abbadia San Salvatore 1, Siena, Italy

**Keywords:** Mt. Amiata, Mercury, Plant uptake, Soils and plants, Bioaccumulation factor, Foliage

## Abstract

The distribution of heavy metals in plants (*Castanea sativa, Sambucus nigra, Verbascum thapsus, Popolus spp., Salix spp., Acer pseudoplatanus, Robinia pseudoacacia)* growing in soils from active and abandoned mining areas is of scientific significance as it allows to recognize their ability to survive in a hostile environment and provide useful indications for phytoremediation operations. In this work, soils from the former Hg-mining area of Abbadia San Salvatore (Tuscany, Central Italy) were analyzed for total, leached Hg, % of organic and inorganic-related Hg. The dehydrogenase enzyme activity (DHA) was also measured with the aim to evaluate the status of the soil, being characterized by high Hg contents (up to 1068 mg kg^−1^). Eventually, the concentration of Hg in the different parts of the plants growing on these soils was also determined. Most studied soils were dominated by inorganic Hg (up to 92%) while the DHA concentrations were < 151 µg TPF g^−1^ day^−1^, suggesting that the presence of Hg is not significantly affecting the enzymatic soil activity. This is also supported by the bioaccumulation factor (BF), being predominantly characterized by values < 1. *Sambucus nigra* and *Verbascum thapsus* had the highest Hg contents (39.42 and 54.54 mg kg^−1^, respectively). The plant leaves appear to be the main pathways of Hg uptake, as also observed in other mining areas, e.g., Almadèn (Spain), indicating that particulate-Hg and Hg^0^ are the main forms entering the plant system, the latter derived by the GEM emitted by both the edifices hosting the roasting furnaces and the soils themselves.

**Supplementary Information:**

The online version contains supplementary material available at 10.1007/s10653-023-01739-w.

## Introduction

Since 2011 the world-class mercury (Hg) mining district, centered in the Municipality of Abbadia San Salvatore (Tuscany, Italy) and located in the eastern part of the Mt. Amiata silicic volcanic complex (Conticelli et al., 2004, 2015; Ferrari et al., 1996; Laurenzi et al., 2015), has been undergoing remediation operations. Since the nineteenth century, Abbadia San Salvatore (ASS) has been one of the most important sites for the exploitation of cinnabar and production of liquid Hg. The ore deposit was indeed excavated, dried and roasted and, through a condensation system, liquid Hg was produced. Through the years, different furnaces were used: from those fed by wood to Spirek-Cermak, from the Gould Pacific to Nesa. It has been estimated that about 70% of the total production of Hg from the whole Mt. Amiata mines was from ASS (Cipriani & Tanelli, 1983). The mining structures of ASS produced more than 100,000 tons of liquid Hg and about 10,000 tons were dispersed into the environment (Bacci et al., 2000; Vaselli et al., 2015). Past and recent geochemical investigations (e.g., Vaselli et al., 2019 and references therein) carried out in soils, waters and air inside the mining area and surroundings have highlighted that most environmental matrices have Hg concentrations much higher than those regulated by the European legislative levels.

According to Jiskra et al. (2018) and Zhou and Obrist (2021), the atmospheric Hg assimilation from vegetation and its transfer to soil and water via throughfall and litterfall are the main sources of Hg in the terrestrial ecosystem. According to Higueras et al. (2012), Barquero et al. (2019) and Naharro et al. (2019), plants growing on Hg-rich soils tend to uptake Hg as Hg^0^ is released from the soils to the atmosphere via stomata whereas less probable is the vehiculation of Hg via plant roots (Naharro et al., 2019 and references therein).

Concentrations of Hg-bearing organic forms in terrestrial environments are, generally speaking, at least one order of magnitude lower than those related to inorganic Hg, although the accumulation of Hg in food webs is still not clear (Bailey et al., 2002; Zhang et al., 2022). The primary warning system of alterations in terms of soil health and quality are soil enzymes, which can be considered potential bio-indicators to assess the soil health status (Datta et al., 2021 and reference therein). Among all soil enzymes, dehydrogenase (DHA), alkaline phosphate (ALP) and urease (UR) are sensitive to both potentially toxic elements (PTEs) and minimal environmental changes (e.g., Araujo et al., 2015; Datta et al., 2021; Elmayel et al., 2020; Gallego et al., 2021; Oliviera & Pampulha, 2006; Santos et al., 2011). In particular, DHA resides within all living microbial cells and, for this reason, it is considered a critical indicator of the enzymatic and microbial oxidative activities of soils (Furtak & Gajda, 2017; Tan et al., 2017; Wolinska & Stepniewska, 2012; Yuan & Yue, 2012). According to Tazisong et al. (2012) and Mahbub et al. (2016), scarce information is presently available about the biological functionality of Hg in plants. Nevertheless, this element has the ability to decrease enzyme activity by binding to both the protein's -SH residues and the active sites of enzyme and protein substrate complexes, or substituting metal cofactors such as Ca or Mg and altering the structure of these compounds (Mahbub et al., 2016; Tazisong et al., 2012). The environmental footprint resulting from the mining activity in the Mt. Amiata and, particularly, at ASS is evidenced by numerous scientific investigations published in the last two decades (e.g., Bacci et al., 2000; Meloni et al., 2021; Rimondi et al., 2012, 2014a, 2014b a, b, 2015) focused on the distribution of Hg in air, soil, vegetation and aquatic compartments (e.g., Chiarantini et al., 2016, 2017; Ferrara et al., 1998; Lazzaroni et al., 2022; Vaselli et al., 2013, 2015, 2021). Previous works, conducted in the mining area of ASS (Chiarantini et al., 2016) and Almadén (Spain) (e.g., Barquero et al., 2019), the most famous Hg-district in the world, investigated the relationship between airborne Hg (Hg^0^) and the vascular vegetation as well as the interaction between DHA and Hg concentrations in soils, evidencing that high Hg contents in soils do not affect DHA (Campos et al., 2018). Barquero et al. (2019) and Chiarantini et al. (2016) analyzed pine trees belonging to the *Pinus Nigra* and *Pinus Pinea* families, respectively.

In this paper, the distribution of Hg between soils and the most common plants growing in the former mining area of ASS and selected parts of them (e.g., roots, trunks, leaves) is presented and discussed in order to understand at which level they are either stressed by or recalcitrant to high Hg soil contents. Moreover, for the first time in this area, the possible interaction between the enzyme DHA and Hg in the pedological cover is evaluated.

### The study area: ASS mine

**Fig. 1:**
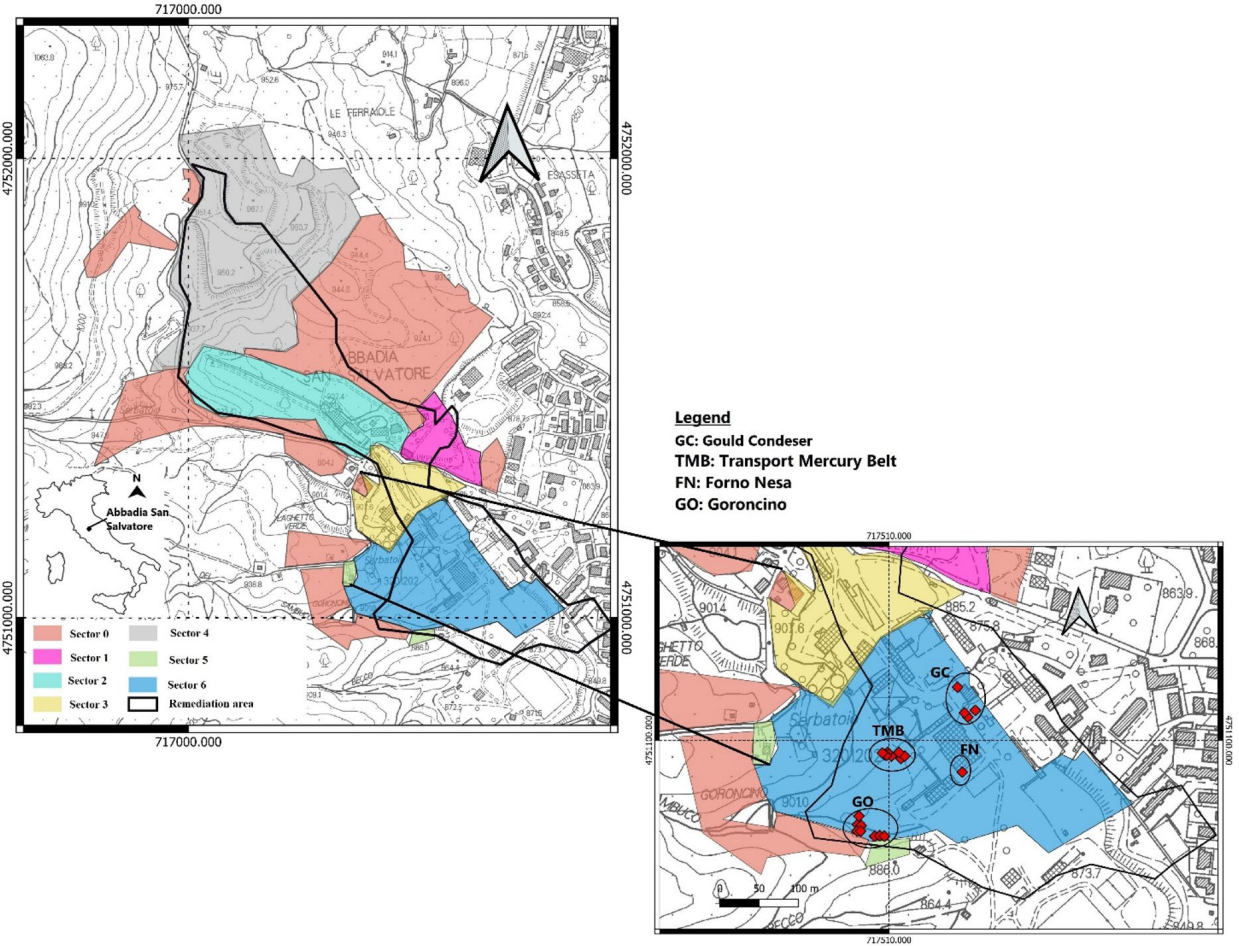
**a** The reclamation area of ASS mine (modified from Vaselli et al., 2017**a** scale 1:15.000. **b** Inset of Sector 6 (study area) with the four sampling areas from where soils and plants were collected. The IDs of the soil and plant samples collected from each area are listed in Supplementary Material, Table S1

The ASS mining area is located in the NW part of the local urban center. The main deposits consisted of cinnabar (HgS), with smaller amounts of pyrite, marcasite, orpiment and realgar (Rimondi et al., 2015). All the exploitation was underground and the galleries reached 400 m below the ground level (e.g., Lazzaroni et al., 2022). Mercury production began in 1899, following the abandonment of the area for about 2000 years since Etruscans and Romans used cinnabar as pigment (Botticelli, 2019; Fantoni et al., 2022). The old mining area included a large wood deposit for the old furnaces, some ancient dryers, and a few tanks that were used to cool gaseous Hg as it passed through the condensers. New dryers, belt conveyor systems and horizontal (Gould) and vertical (Nesa) furnaces were installed in the following years, along with more efficient condensation systems. In 1976, the production activity at ASS dramatically slowed down since the exploitation of Hg was not economically sustainable and its use became conspicuously harmful and toxic. In 1982, the whole mining plant was definitively shut down. In the 1990s, ENI (Ente Nazionale Idrocarburi)—AGIP (Azienda Generale Italiana Petroli) Unit—proposed a reclamation project to permanently close the former mining and industrial activity. In 2008, an agreement between the Municipality of ASS and the former owner of the mining concession (ENI-AGIP) was signed. The mining concession and the reclamation project were thus transferred to the public administration. The ENI project was fully revised by the local municipality and the reclamation operations were then addressed to the environmental restoration of the mining areas and buildings for museum and public utility purposes (Vaselli et al., 2019). Consequently, the whole mining concession was divided into seven different units (Fig. 1a), including the reclamation of about 65 ha (black contour in Fig. 1a): Sectors 0 and 1 are the sites where Hg was found at low concentrations; Sectors 2 and 3 host the miners’ and managers’ buildings, the mining equipment grounding area, the conveyor belts, and the Garibaldi shaft, and the old furnaces, dryers, and condensers, respectively; Sector 4, namely ‘Le Lame,’ consists of the most important mining dump of ASS mining area and covers a surface of 120,000 m^2^ (e.g., Meloni et al., 2021 and references therein); Sector 5 contains the armory and the watchmen’s house; Sector 6, which is the most contaminated site among the 6 sectors, hosts the main critical mining facilities, e.g., Nesa and Gould furnaces, old and new driers, condensation tubing systems, pigment preparation edifices and collection tanks of liquid Hg (Vaselli et al., 2017a).

The reclamation of the ASS mine began in 2013 with the construction of the bypass channel to minimize the water–rock interaction between rainwater and Hg-contaminated soils and the removal of Eternit® (a mixture of cement and asbestos fibers) from the roofs (Vaselli et al., 2015). Remediation is still ongoing and the clean-up of Sector 6 has recently begun. All soil and plant samples analyzed for this work were collected in February of 2018 from Sector 6, when the reclamation had not still started. The sampling area can be divided into: 1) Transport Mercury Belt (TMB), near the furnaces where cinnabar was roasted to extract gaseous Hg; 2) Forno Nesa (FN); 3) Goroncino (GO), where tailings from the local and other Mt. Amiata Hg-mines were stored, and 4) Gould Condensers (GC), where the gaseous Hg was cooled down after the roasting process (Fig. 1b).

## Materials and methods

Twenty-four plants (eight different species: *Castanea sativa*, *Sambucus nigra*, *Verbascum thapsus*, *Popolus spp.*, *Salix spp.*, *Acer pseudoplatanus*, *Robinia pseudoacacia* and *Cytisus scoparius*) and related soils were collected. Soils were sampled at a depth between 15 and 20 cm in order to analyze the total Hg distribution between roots and soils (Johnson et al., 2005). Two years before the sampling date, a complete vegetation clearcutting was carried out. Thus, the age of the sampled plants was known. It was decided to divide the plant samples into bark, internal and external roots, bark and internal trunks, medulla (when the trunk or root presented it) and foliage. Table S1 (Supplementary Material) reports the geographic coordinates in WGS84-UTM 32N, the soil IDs, the sampling location, the Latin name of the sampled plants and the parts into which each plant was divided and analyzed. For each plant sample, the soil particles were brushed off manually. Subsequently, the plant samples were washed in an ultrasonic bath until the MilliQ water was clean. Then, they were heated in an oven for at least three days at 35 °C. Eventually, each plant portion was ground into small pieces using a coffee grinder.

The soil samples were stored in an oven at a temperature of 35 °C (to prevent any Hg^0^ loss) until they were dried, and then sieved at 2 mm. A representative aliquot of the < 2-mm samples (about 100 g) was pulverized in a planetary mill (Pulverisette 5) equipped with agate mortars and balls. The pH values for each soil were determined following the UNI_EN 15933:2012 norm using a multi-probe Hanna HI98194.

Mercury concentrations in soils and plants were determined by a Lumex RA 915 + (Atomic Absorption Spectrometry with Zeeman effect) instrument (Sholupov et al., 2004), equipped with a Pyro-915 + device. Additionally, the percentage of Hg related to the organic (bound to humic acids) and inorganic fractions was also estimated (Rumayor et al., 2016). In fact, it must be taken into account that biological samples burn at 275–290 °C. At this temperature interval, all Hg bound to humic acid compounds decomposes and releases Hg^0^. The instrument was calibrated using Montana soil 2710A and the error was < 10%.

The analysis of DHA was aimed at quantifying the amount of organic activity in soils. A mixture consisting of 0.15 mL of distilled water, 0.015 g of CaCO_3_ and 0.25 mL of 3% (m/V) of triphenyl tetrazolium chloride (TTC, C_19_H_15_ClN_4_) was prepared and it was left reacting with 1.5 g of soil. The samples were then introduced in an incubator at 37 °C for 24 h (Casida et al., 1964; Montejo et al., 2012) and eventually cooled in a freezer for 10 min to stop the reaction. Subsequently, 5 mL of methanol was added to extract the formed TTC. The colored extract was measured at 485 nm by molecular spectrophotometry. All these analyses were carried out at the IGeA Laboratories (Instituto de Geología Aplicada, University of Castilla La Mancha) in Almadén (Ciudad Real, Spain).

The leachable Hg was determined by ICP-MS using the USEPA 1312 method (USEPA, 1994) at the CSA Laboratories in Rimini (Italy) in order to simulate the fraction of soluble Hg in acid mine drainage conditions. This extraction entails mixing 5 g of soil sample with 100 mL of the EPA solution. A 60/40 combination of H_2_SO_4_ and HNO_3_ was added to 2 L of distilled water to create the EPA solution (pH of 4.5 ± 0.05). After mixing the soil with the EPA solution, the samples were heated in a stirrer thermostatic bath for 18 h at 30 revolutions per minute. Water was added to maintain a constant temperature of 25 °C. After 18 h, the samples were filtered using glass fiber filters with a 0.45 µm pore size.

All statistical calculations (e.g., minimum, maximum, average, Pearson correlation) were performed with R and R studio (R Core Team, 2021), while all graphics were realized with Origin 2021 b. When the chemical data were below the Limit Of Quantification (LOQ) (< 0.0005 mg kg^−1^ for Hg, and < 0.001 for BF), they were replaced with 2/3 of the LOQ itself (Gozzi et al., 2021).

### Hg bioaccumulation factor (BF)

According to Campos et al. (2018), the bioaccumulation factor reflects the bioavailability of Hg in plants. The Bioaccumulation Factor (BF) is a parameter used to measure the transfer capacity of PTEs from soil to plant (Wang et al., 2016). In this study, BF was calculated as the ratio between the Hg concentration in the different parts of the plants and the soil leachable Hg content (Eq. 1):

1 $$BF=\frac{{Hg}_{p}}{ {Hg}_{SL}}$$

where Hg_p_ is the Hg concentration in the selected part of each plant while Hg_SL_ is the leachable Hg content recalculated to the amount of leached soil. Differently to other elements, in this case, BF expresses the uptake of the element via indirect ways. Basically, it can be referred to the Hg uptake and bioaccumulation in the different analyzed plant parts.

## Results

### Hg concentration and DHA in topsoils

The main descriptive statistics of the pH, total Hg, leached Hg, soil leachable Hg, % Hg inorganic, % Hg organic and DHA values in the studied soils (e.g., number of observations, minimum, maximum, mean, median, standard deviation) are summarized in Table 1. The full dataset is reported in Table S2 (Supplementary Material).

**Table 1:** Number of observations (N.obs.), minimum, maximum, average, median and standard deviation (SD) of pH, total Hg (in mg kg^−1^), leached Hg (in µg L^−1^), soil leachable Hg (in mg kg^−1^), inorganic Hg (in %), organic Hg (in %), and DHA (in µg TPF g^−1^ day^−1^). n.d.: no detected. The full data are reported in Supplementary Material-Table S2

	N. obs	Minimum	Maximum	Average	Median	SD
pH	24	7.9	8.8	8.5	8.5	0.2
Total Hg	24	2	1068	462	503	318
Leached Hg	24	< 0.1	20	3	0.3	5
Soil Leachable Hg	24	n.d	8.56	1.15	0.12	2.12
Inorganic Hg	24	n.d	94	59	56	22
Organic Hg	24	n.d	74	41	44	22
DHA	24	< 1	166	54	37	49

The pH values of the ASS mining soils are mostly alkaline. Total Hg in soils reached an average value of 462 mg kg^−1^, with minimum and maximum concentrations of 2 and 1068 mg kg^−1^, respectively. The lowest content of Hg in the leached soil was below the instrumental detection limit (0.1 µg L^−1^, sample ASS2) whereas the highest concentration was 20.4 µg L^−1^ (ASS14), which corresponds to a soil leachable Hg of 8.56 mg kg^−1^. The percentage of inorganic and organic Hg in soils, with the exception of sample ASS1 (where it was not possible to measure the relative percentage), was up to 93.9% and 73.5%, respectively. Regarding DHA, only the sample ASS2 had concentration < 1 µg TPF g^−1^ day^−1^ whereas the maximum value was 166.0 µg TPF g^−1^ day^−1^ (ASS8b).

### Hg in plants

The minimum, maximum of BF values and minimum, maximum and median of Hg concentrations in the eight types of plants collected from each study area, without distinguishing the different plant portions, are summarized in Table 2. All data are listed in Table S3 (Supplementary Material).

**Table 2:** Maximum (Max), minimum (Min) and average of Hg concentrations (mg kg^−1^), and maximum (Max), minimum (Min) and average of bioaccumulation factor (BF) values of the eight plant species collected within the former mining area according to the different sampling sites: TMB, FN, GO and CG. Full data are shown in Table S3 (Supplementary Material)

	TMB						FN					
Plants	Hg (mg kg^−1^)			BF			Hg (mg kg^−1^)			BF		
	Max	Min	Average	Max	Min	Average	Max	Min	Average	Max	Min	Average
*Cytisus Scoparius*	< 0.0005	14.36	2.19	0.059	< 0.001	0.018	–	–	–	–	–	–
*Popolus spp.*	0.28	16.86	4.05	0.13	0.002	0.032	–	–	–	–	–	–
*Robinia pseudoacacia*	0.52	0.68	0.60	< 0.001	< 0.001	< 0.001	0.16	18.4	4.96	0.219	0.001	0.0586
*Castanea Sativa*	1.08	23.75	8.23	0.565	0.025	0.196	–	–	–	–	–	–
*Sambucus nigra*	3.75	39.42	15.23	0.938	0.089	0.362	–	–	–	–	–	–
*Verbascum Thapsus*	0.04	23.68	12.80	0.563	0.001	0.304	–	–	–	–	–	–
*Salix spp.*	–	–	–	–	–	–	–	–	–	–	–	–
*Acer pseudoplatanus*	–	–	–	–	–	–	–	–	–	–	–	–
	GO						CG					
Plants	Hg (mg kg^−1^)			BF			Hg (mg kg^−1^)			BF		
	Max	Min	Average	Max	Min	Average	Max	Min	Average	Max	Min	Average
*Cytisus Scoparius*	0.12	6.7	2.838	0.039	< 0.001	0.016	–	–	–	–	–	–
*Popolus spp.*	0.34	0.56	0.45	0.018	0.003	0.011	–	–	–	–	–	–
*Robinia pseudoacacia*	0.05	9.25	2.193	0.073	< 0.001	0.017	0.23	14.58	7.405	0.015	< 0.001	0.0078
*Castanea Sativa*	0.07	5.3	2.258	< 0.001	< 0.001	< 0.001	–	–	–	–	–	–
*Sambucus nigra*	0.26	2.05	0.917	< 0.001	< 0.001	< 0.001	0.92	8.08	3.42	0.003	< 0.001	0.0014
*Verbascum Thapsus*	0.43	54.55	21.903	0.649	0.005	0.260	13	37.74	24.71	0.024	0.008	0.0157
*Salix spp.*	0.09	6.17	1.457	0.146	0.002	0.034	–	–	–	–	–	–
*Acer pseudoplatanus*	0.1	0.67	0.385	0.008	0.001	0.0045	0.38	3.13	1.755	0.001	< 0.001	0.0008

According to Table 2, *Verbascum thapsus* is the plant where the highest Hg concentration (54.54 mg kg^−1^) was measured, whereas the lowest content pertained to *Acer pseudoplatanus* (3.13 mg kg^−1^). As far as the BF values are concerned, *Sambucus nigra* and *Verbascum thapsus* are the two plant species able to accumulate the highest amount of Hg (max. values BF: 0.93 and 0.64, respectively) when no distinction on single plant parts is considered. The remaining plants show a maximum BF value of < 0.60.

## Discussion

Regarding the total Hg distribution in soils is concerned, the GC zone results are the area with the highest concentration of Hg, followed by TMB > GO > FN (Fig. 1b). No significant correlation between total and leached Hg is observed, indicating that Hg is heterogeneously distributed in the investigated soils, being likely related to different sources. In contrast to Campos et al. (2018), who reported a correlation between total Hg and soil leached Hg of 0.79, and a correlation between soil leached Hg and humic acid Hg of 0.65, in this work soil leached Hg do not show any correlation between the analyzed Hg species. The topsoils are indeed affected by the presence of anthropogenic materials. In the past, in some portions of the former mining area, including the sites where the soil samples and plants were collected, post-roasting and anthropic manmade (e.g., bricks, tiles, fragments of concrete) materials were used to fill a small paleo-valley positioned in front of the edifice hosting the Gould and Nesa furnaces (Fig. 1b) (Vaselli et al., 2015).

**Fig. 2:**
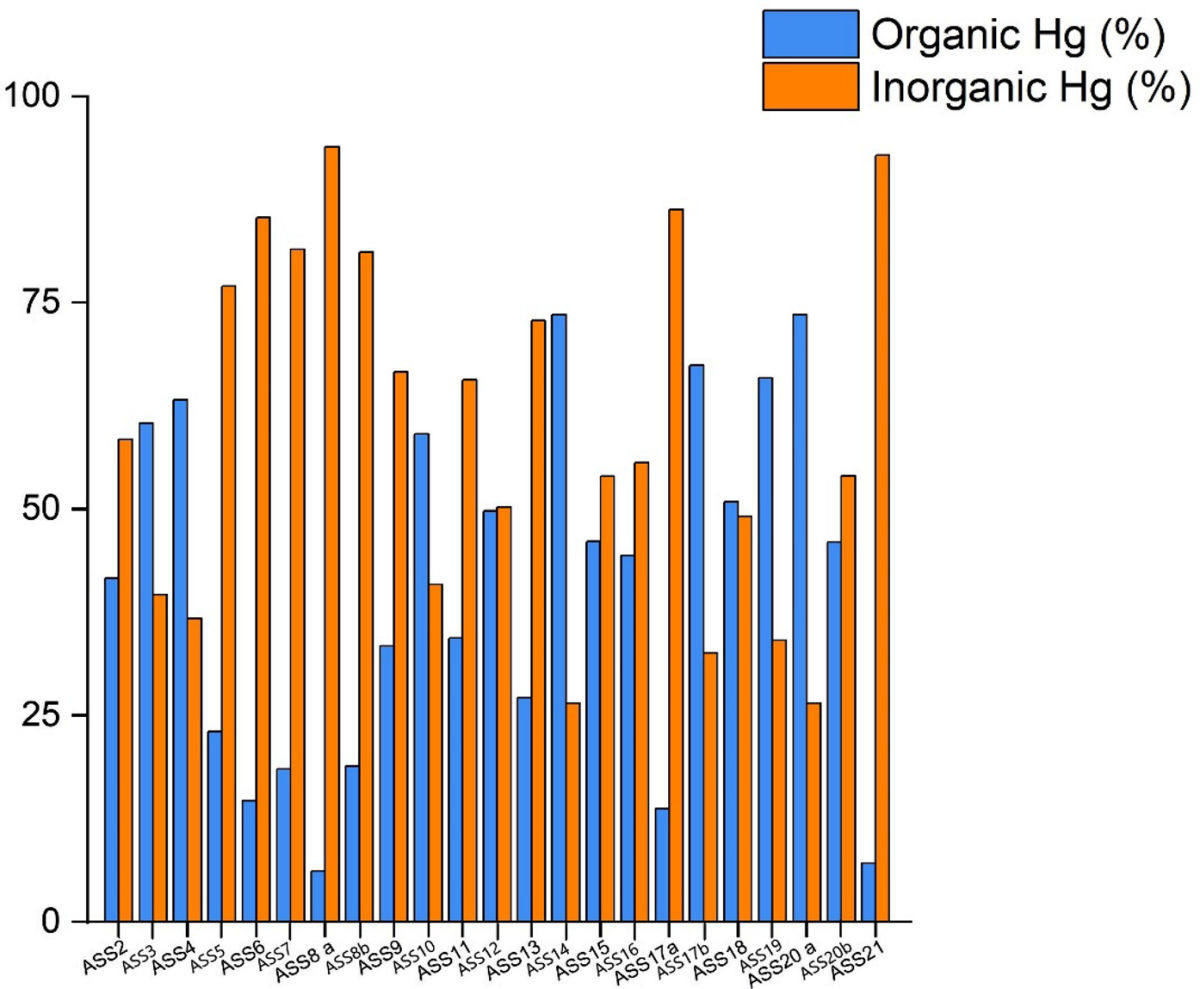
Bar chart plot of organic and inorganic Hg (in %) in the soil samples

The thermal speciation data evidenced that most Hg is inorganic although eight topsoils (i.e., ASS3, ASS4, ASS10, ASS14, ASS17b, ASS18, ASS19, and ASS20a) have a percentage of organic-related Hg that prevails over that related to inorganic Hg, as evidenced in the bar plot chart of Fig. 2.

**Fig. 3:**
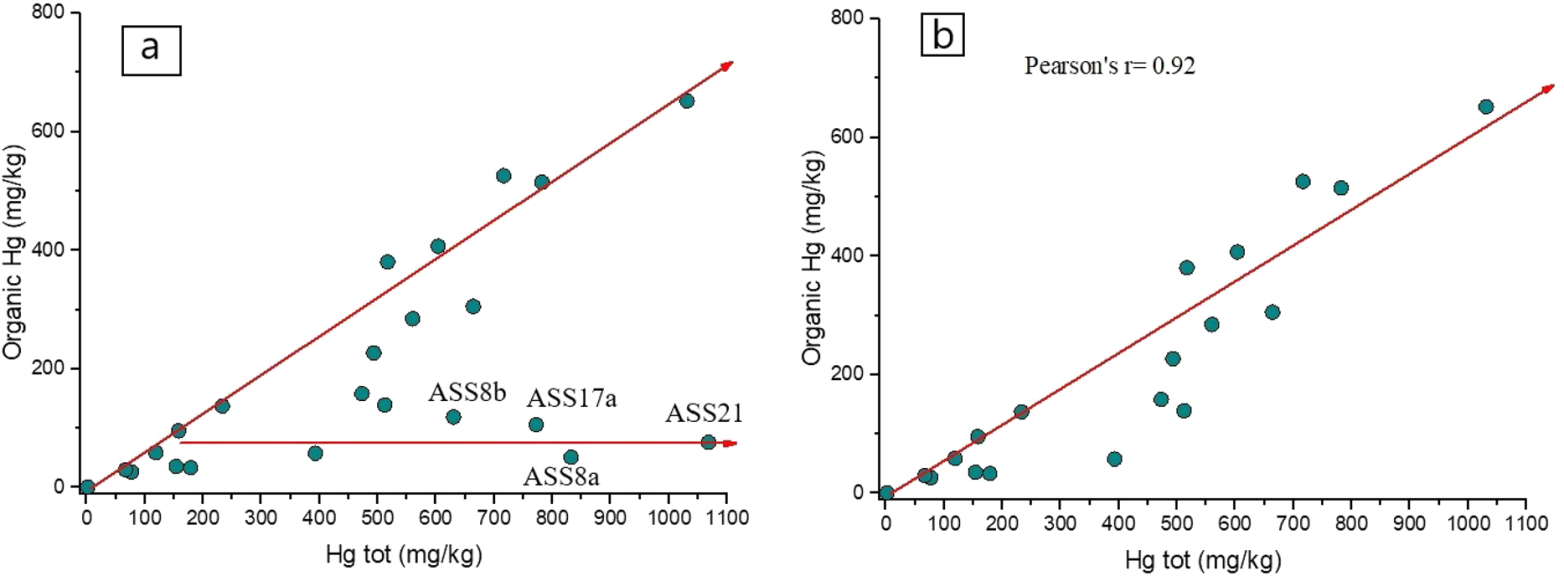
Binary diagram between organic-related Hg vs. Total Hg (in mg kg^−1^) with **a** and without sampled ASS8a,b, ASS17a, ASS21 **b**. The organic Hg concentrations are those computed by thermal speciation

**Fig. 4:**
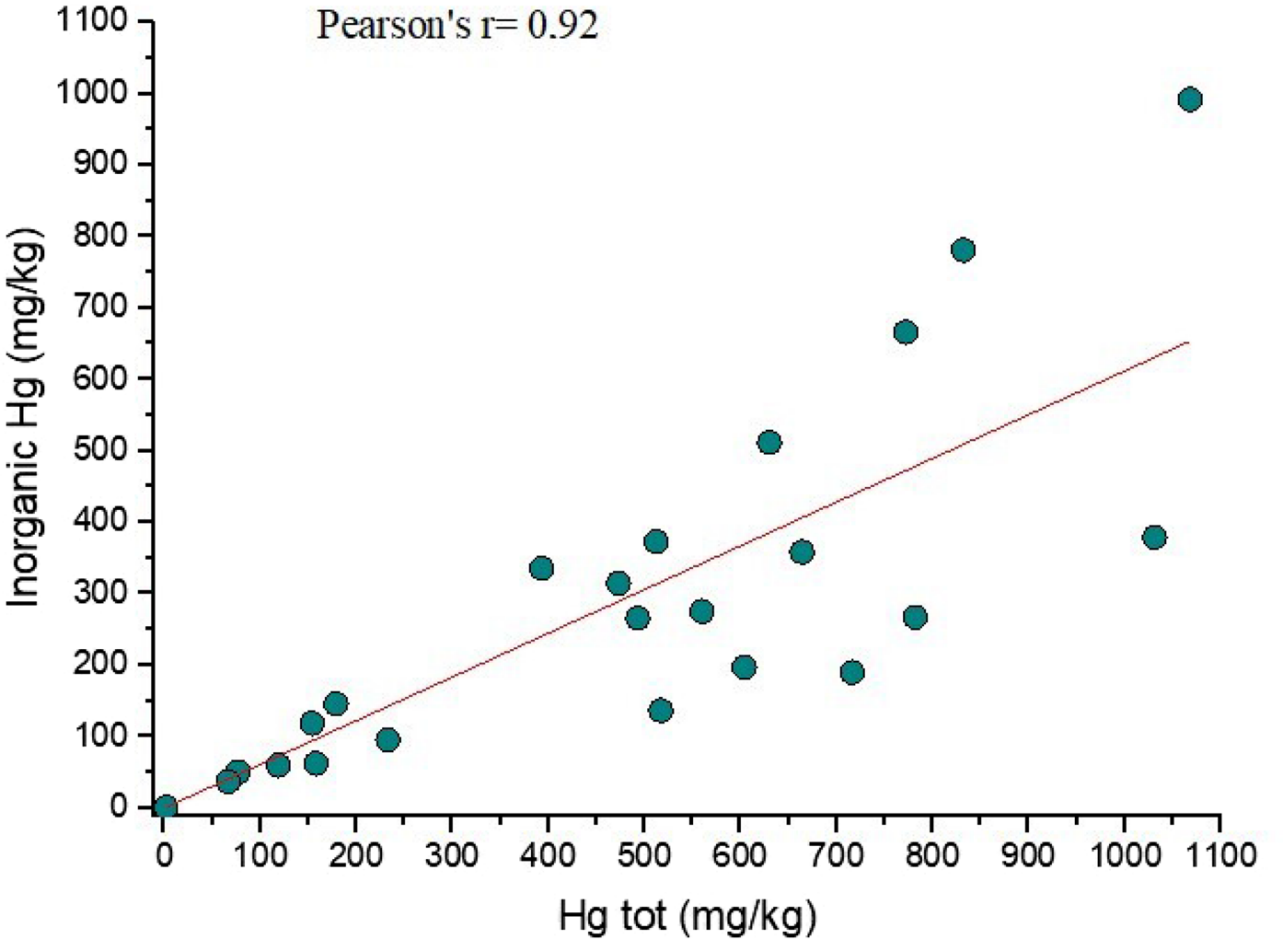
Binary diagram between inorganic-related Hg vs. Total Hg (in mg kg^−1^). The inorganic Hg concentrations are those computed by thermal speciation

In this study, as well as in the Almadenejos metallurgical precinct (Almadén, Spain; Campos et al. (2018), a positive correlation was found between total Hg and that corresponding to the fractions identified by thermal speciation, i.e., Hg-related humic acids and inorganic Hg (Fig. 3a, b and Fig. 4). In Fig. 3a, two distinct trends can be observed. The first one corresponds to a positive correlation between total Hg and organic Hg whereas the second trend is mainly delineated by four samples (ASS8a, ASS8b, ASS17a and ASS21), which are characterized by an increasing concentration of the computed inorganic Hg (up to > 80%) while organic Hg maintains almost unchanged. This likely implies that these soil samples are possibly indicating the presence of higher contents of residual mining materials. When these four samples are not considered, the correlation between the two parameters significantly increases since a Pearson coefficient of 0.92 (Fig. 3b) was computed. A similar positive correlation (*r* = 0.92) is also obtained when total Hg is plotted *vs.* inorganic-related Hg (Fig. 4). This interdependence indicates that the fractionation of Hg compounds in the soils of the ASS mine is a distinct process unaffected by the relative position of the samples, the amount of organic matter present, and the activity due to enzymatic processes, as also reported by Campos et al. (2018). It is be noticed that the soil samples located in GO (Fig. 1b and Table S1) are more enriched in organic-related Hg with respect to those collected close to the mining facilities, suggesting that the proximity to the machineries where liquid Hg was produced affected the soil matrix.

**Fig. 5:**
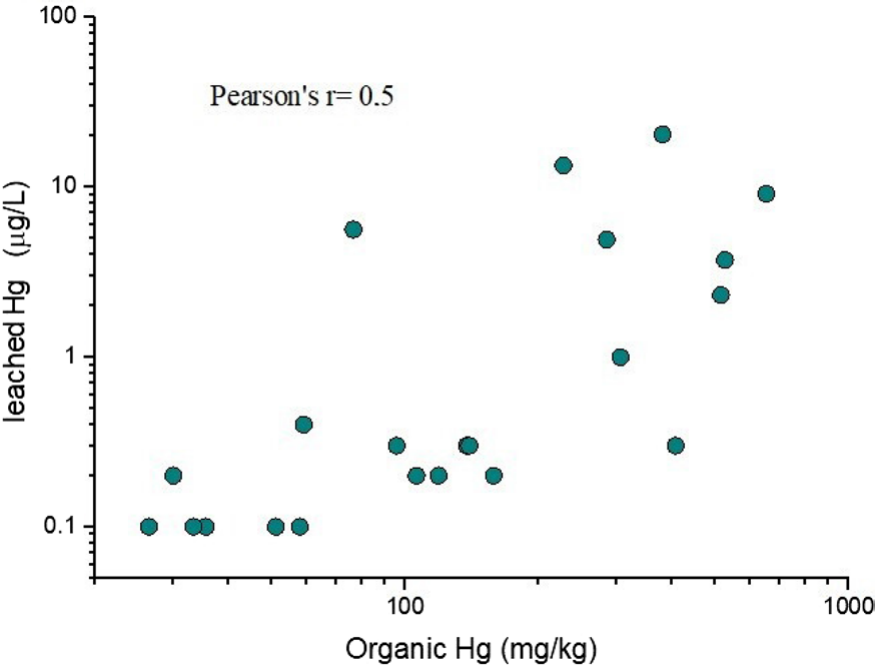
Log-scale binary diagram between leached (in µg L^−1^) and organic-related Hg. The organic Hg concentrations are those computed by thermal speciation

In addition, a second, though weaker, correlation (*r* = 0.5) between leachable Hg (in µg L^−1^) and organic-related Hg (in mg.

kg^−1^) is reported in Fig. 5. According to Campos et al. (2018), the most labile species of Hg are those containing organic Hg. However, it is pointed out that the high concentration of leached Hg (up to 20 µg L^−1^) can also be released by solid phases and is not necessarily only related to organic Hg.

The measured DHA contents have an average value of 53.7 µg TPF g^−1^ day^−1^. This value is markedly lower than that measured by Campos et al. (2018) in the Almadenejos soils and approaches reported by Hinojosa et al. (2004) for soils polluted by heavy metals (average: 70 µg TPF g^−1^ day^−1^).

**Fig. 6:**
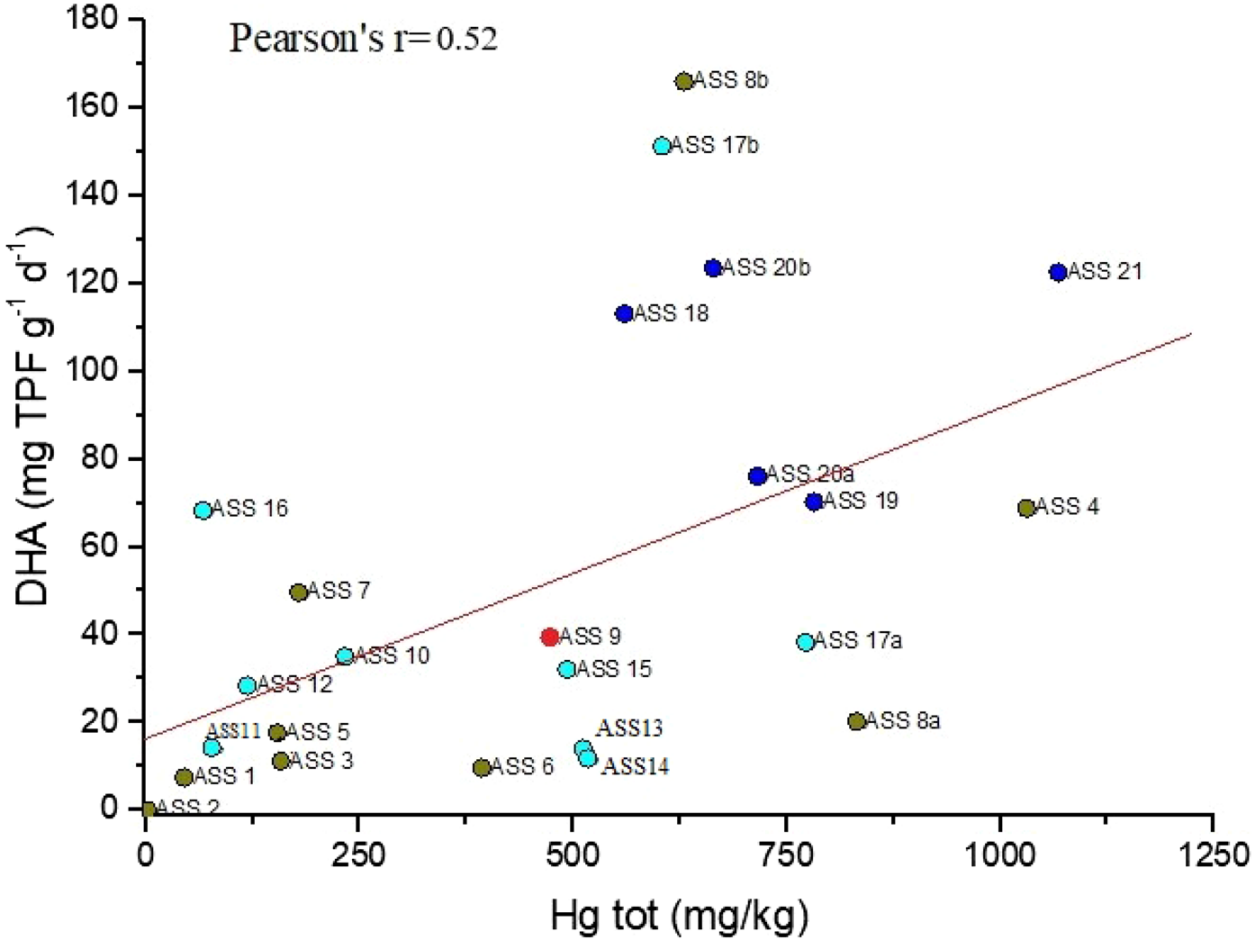
Scatterplot of DHA (mg TPF g^−1^d^−1^) vs. Hg (mg kg^−1^) in the soils from the ASS mining area. Blue circles: samples from CG, red circles: samples from FN, cyan circle: samples from GO and dark yellow circle: samples from TMB

The concentration of DHA in the soils collected from the mining and production area appears to be even lower than those measured by Hinojosa et al. (2004) in the reclaimed site of the Aznalcollar mine (SW Spain). According to Pan and Yu (2011), the presence of PTEs in soils can have negative effects on enzymatic activity, affecting either the enzyme–substrate complexation or the structure of the amino acids. In this case, the total Hg *vs.* DHA enzyme diagram (Fig. 6) shows a poor correlation (Pearson correlation *r* = 0.52, *p* < 0.05) with scatter distribution between the two parameters, suggesting that the presence of Hg, independently by its speciation, is not able to affect the microbial activity in the ASS soils, similarly to what observed by Campos et al. (2018) for the Hg-rich soils from Almadenejos.

Considering the metal and metalloid concentrations usually found in the AAS ore deposits and the surrounding Hg-mining areas (Meloni et al., 2021; Rimondi et al., 2014a), the analytical spectrum should be enlarged to evidence whether the enzymatic activity may be jeopardized by other PTEs (e.g., As and Sb).

### Hg and BF in plants

**Fig. 7:**
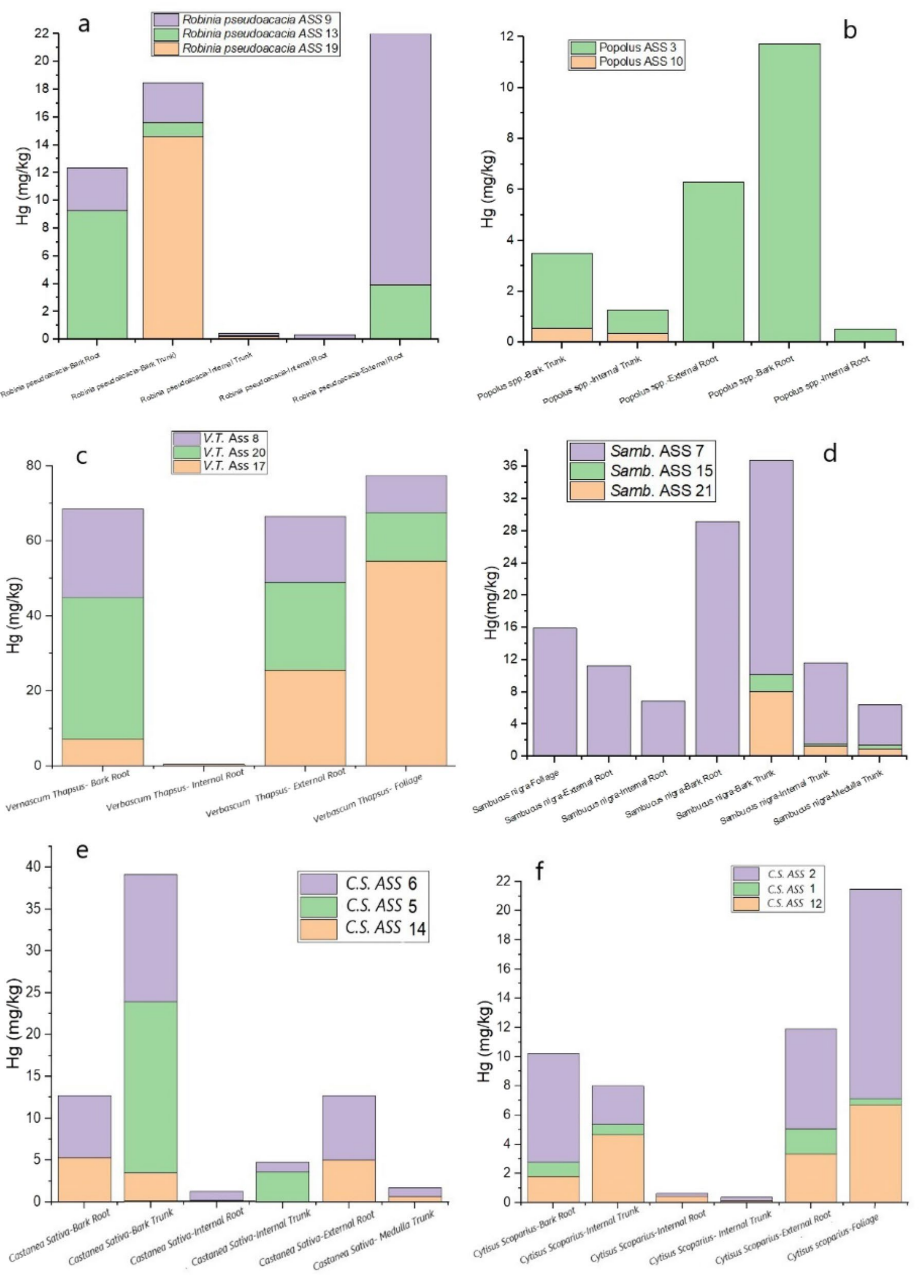
Bar graphs of the Hg amount in each analyzed portion of *Robinia pseudoacacia* **a***, Popolus spp.* **b**, *Verbascum thapsus* **c** *Sambucus nigra* **d**, *Castanea sativa* **e**, and *Cytisus scoparius* (f)

The bar graphs in Fig. 7 depict the Hg distribution in each portion of the sampled plants, except for *Acer pseudoplatanus* and *Salix spp.*, for which only one sample (bark trunk and root, respectively) was collected. *Robinia pseudoacacia*, *Sambucus nigra*, *Castanea sativa* and *Popolus spp.* are characterized by the highest Hg concentrations in the roots, as well as *Salix spp.*, while the bark trunk of *Acer* is enriched in Hg. Different is the behavior of *Cytisus scoparius* and *Verbascum thapsus* as Hg is found in high contents in the foliage. Notably, is the fact that the highest Hg concentrations are related to the leaves of *Cytisus scoparius*, located in the TMB zone (Fig. 1), where gaseous elemental Hg in the atmosphere was found almost constantly up to 50,000 ng m^−3^ or even higher (Vaselli et al., 2013).

This is likely related to the fact that the leaf system is one of the main pathways of Hg uptake due to both dry deposition, as also suggested by Chiarantini et al. (2016) and Campos et al. (2018), as well as gaseous Hg from Hg-rich environments (such as that recorded in the air nearby the mining machineries and furnaces) and diffuse Hg from soil, although no Hg flux measurements are presently available.

The high Hg concentrations detected in the leaves of *Verbascum thapsus* (related to ASS 17 soil), collected from GO (Fig. 1b), can be explained by the main winds at ASS that blow from NNE/NE (https://www.meteoblue.com/it/tempo/historyclimate/climatemodelled/abbadia-san-salvatore_italia_3183581), thus, favoring the deposition of the Hg-rich atmospheric particulate and atmospheric Hg from TMB to GO (Fig. 1b). Therefore, according to the investigation on the different parts of plants analyzed in this study (Fig. 7), foliage is likely the main mechanism of Hg-uptake that can be invoked for the ASS plants, thus confirming previous investigations, e.g., Naharro et al., (2019). Occasionally, roots seem to play a role in the Hg-uptake. However, further analyses on the leaf apparatus for those plants where the foliage was not collected are necessary.

**Fig. 8:**
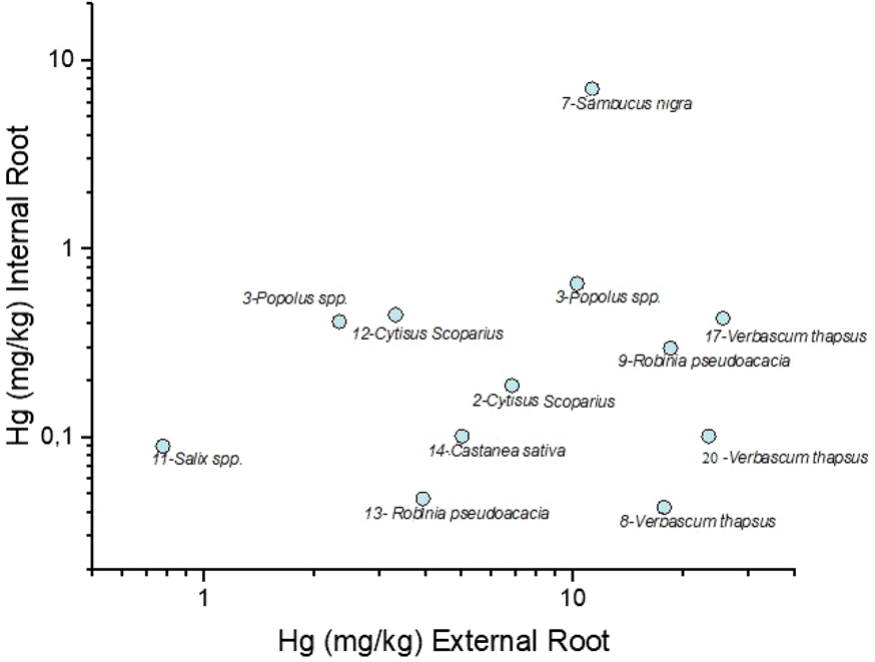
Scatterplot of Hg (mg kg^−1^) in external root vs. Hg (mg kg.^−1^) in internal root of the plant samples in bi-logarithmic scale. The numbers before the plant name correspond to the related soil (see Table S1)

The distribution of Hg between external and internal roots is reported in Fig. 8. All the studied root samples indicate that Hg concentration increases, as expected, in the external roots, with the exception of *Sambucus nigra*, showing an external root/internal ratio of about 1.7, i.e., more than one order of magnitude lower than those recorded for those samples characterized by Hg content > 20 mg kg^−1^. To the best of our knowledge, few are the studies related to the partitioning of Hg between internal and external roots and this calls for more detailed investigations.

According to Hussain et al. (2022), the BF is a partitioning coefficient that mimics the ability of plants to absorb PTEs and, in this study, it was applied to the concentration of Hg in each part of the analyzed plants and that related to the soil leachable Hg. The BF values are highly variable when the different plant sectors are considered (Table S3). All BF values are < 1 (Table S3), but BF > 0.6 values correspond to the bark trunk and bark root of *Sambucus nigra*, and the leaves of *Verbascum thapsus* (0.63, 0.93, and 0.65 respectively). The BF values in the leaves of *Verbasum thapsus* seem to confirm that the leaves are likely the main path of Hg-uptake by plants. On the other hand, the relatively high BF values measured in the bark trunk and bark roots of *Sambucus nigra* are possibly due to the difficulty in efficiently and completely removing all the soil-related particles during cleaning. This means that the concentration of mercury in the outermost part of roots and trunk is likely affected by the presence of soil material.

## Conclusions

In this study, the distribution of Hg in soils and plants growing in the former mining area of Abbadia San Salvatore was investigated and, to the best of our knowledge, DHA concentrations and the BF values were determined for the very first time in one of the most important Hg sites worldwide. The content of Hg in the soils located in the highly Hg contaminated Sector 6 is heterogeneously distributed in the four, high Hg-contaminated, areas from where the plants were collected (Fig. 1b). The highest concentrations were measured close to the edifices hosting the Nesa and Gould furnaces, since here, when the mining works were active, mine tailings and manmade materials related to the mining activity were used to fill a small paleo-valley, likely increasing the original Hg contents. Setting aside eight soils, thermal speciation allowed to recognize that inorganic Hg, presumably associated with cinnabar, was the prevailing species over the organic component. The low DHA concentrations indicates that the area is likely contaminated by PTEs, although investigations are required to evidence their presence. However, the poor, though positive, correlation between total Hg and DHA shows that the Hg compounds do not affect the enzymatic action of DHA and do not inhibit but, conversely, enhance the microbial activity. Mercury concentrations in the studied plants show that the main pathway of Hg-uptake is the leaf system. For those samples where the foliage was not analyzed, roots can apparently play an important role in the uptake of Hg. However, more detailed investigations are needed to fully understand i) the effects on the roots when developing in a Hg-rich pedological environment and ii) the partitioning of Hg between external and internal roots. The BF is < 1 in all samples, indicating a weak soil to plant translocation of mercury. In order to avoid possible errors in computing BF calculation, more careful and repeated washing of the different parts of the plants (especially those parts most in contact with the soil) is recommended. According to this study, phytoremediation projects should take into account the ability of *Sambucus nigra* to uptake Hg. A pilot site consisting of a *Sambucus nigra* plantation, positioned in a secure disposal location within the remediation area, could be established to verify whether the Hg removal is effective. Nevertheless, it can be recommended that more indigenous plants should be thoroughly analyzed to verify whether other species may have a stronger Hg adsorption capacity than that of *Sambucus nigra*.

### Supplementary Information

Below is the link to the electronic supplementary material.

Supplementary file1 (DOCX 40 KB)
