# Supplementary material for: Mercury distribution in plants and soils from the former mining area of Abbadia San Salvatore (Tuscany, Central Italy)
Source: Environ Geochem Health. 2023 Aug 30;45(11):8523–38. doi: 10.1007/s10653-023-01739-w (PMC10611595; doi:10.1007/s10653-023-01739-w)
Supplement: Supplementary file 1 — Supplementary file1 (DOCX 40 KB) [file 10653_2023_1739_MOESM1_ESM.docx]

**Mercury distribution in plants and soils from the former mining area of Abbadia San Salvatore (Tuscany, central Italy)**

Federica Meloni^1,2,^*, Alessandro Farieri^1^ , Pablo L. Higueras^3,^ José M. Esbrí^4^, Barbara Nisi ^2^, Jacopo Cabassi^2^, Daniele Rappuoli^5,6^, Orlando Vaselli^1,2,^*

1 Department of Earth Sciences, Via G. La Pira, 4 – 50121 Firenze (Italy); federica.meloni@unifi.it, Alessandro.farieri@stud.unifi.it, orlando.vaselli@unifi.it

2 CNR-IGG Institute of Geosciences and Earth Resources, Via G. La Pira, 4 - 50121 Firenze (Italy); barbara.nisi@igg.cnr.it, jacopo.cabassi@igg.cnr.it

3 Instituto de Geología Aplicada, EIMIA - Pl. Manuel Meca 1 13400 Almadén, Ciudad Real (Spain); Pablo.Higueras@uclm.es

4 Departament of Mineralogy and Petrology, (UCM) C. de José Antonio Novais, 12, 28040 Madrid, Spain

5 Unione dei Comuni Amiata Val d'Orcia, Unità di Bonifica, Via Grossetana 209 - 53025 Piancastagnaio, Siena (Italy); d.rappuoli@uc-amiatavaldorcia.si.it

6 Parco Museo Minerario di Abbadia San Salvatore - Via Suor Gemma, 53021 Abbadia San Salvatore 1, Siena (Italy)

Correspondence: federica.meloni@unifi.it; [orlando.vaselli@unifi.it](mailto:orlando.vaselli@unifi.it)

**Supplementary material**

**Table S1** Geographic coordinates (WGS84-UTM 32N), soil sample ID, Sample Location (see Fig. 1b) plant species and analyzed plant parts

| X Y | | Soil Sample ID | Sample Location | Latin name of Plants | Analyzed plant parts |
| --- | --- | --- | --- | --- | --- |
| 717501 | 4751083 | ASS1 | Transport Mercury Belt (TMB) | *Cytisus Scoparius* | Bark root and trunk, internal root and trunk, external root, foliage |
| 717522 | 4751084 | ASS2 | Transport Mercury Belt (TMB) | *Cytisus Scoparius* | Bark root and trunk, internal root and trunk, external root, foliage |
| 717504 | 4751081 | ASS3 | Transport Mercury Belt (TMB) | *Popolus spp.* | Bark root and trunk, internal root and trunk, external root |
| 717506 | 4751080 | ASS4 | Transport Mercury Belt (TMB) | *Robinia pseudoRobinia pseudoacacia* | Bark and internal trunk |
| 717512 | 4751079 | ASS5 | Transport Mercury Belt (TMB) | *Castanea Sativa* | Bark and internal trunk |
| 717508 | 4751084 | ASS6 | Transport Mercury Belt (TMB) | *Castanea Sativa* | Bark and internal root and trunk, external root, Medulla root and trunk |
| 717525 | 4751076 | ASS7 | Transport Mercury Belt (TMB) | *Sambucus nigra* | Bark root and trunk, internal root and trunk, external root, foliage, Medulla trunk |
| 717530 | 4751080 | ASS8 a/b | Transport Mercury Belt (TMB) | *Verbascum Thapsus* | Bark root and trunk, internal root and trunk, external root |
| 717603 | 4751059 | ASS9 | Forno Nesa (FN) | *Robinia pseudoRobinia pseudoacacia* | Bark root and trunk, internal root and trunk, external root |
| 717469 | 4750993 | ASS10 | Gornocino (GO) | *Popolus spp.* | Bark and internal trunk |
| 717474 | 4750991 | ASS11 | Gornocino (GO) | *Salix spp.* | Bark root and trunk, internal root and trunk, external root |
| 717469 | 4750985 | ASS12 | Gornocino (GO) | *Cytisus Scoparius* | Bark root and trunk, internal root and trunk, external root, foliage |
| 717473 | 4750984 | ASS13 | Gornocino (GO) | *Robinia pseudoRobinia pseudoacacia* | Bark root and trunk, internal root and trunk, external root |
| 717491 | 4750978 | ASS14 | Gornocino (GO) | *Castanea Sativa* | Bark root and trunk, internal root and trunk, external root, medulla trunk |
| 717498 | 4750979 | ASS15 | Gornocino (GO) | *Sambucus nigra* | Bark and internal trunk, medulla trunk |
| 717504 | 4750978 | ASS16 | Gornocino (GO) | *Acer pseudoplatanus* | Bark and internal trunk |
| 717471 | 4751003 | ASS17a/b | Gornocino (GO) | *Verbascum Thapsus* | Bark, internal and external root, foliage |
| 717610 | 4751129 | ASS18 | Gould Condensers (GC) | *Acer pseudoplatanus* | Bark and internal trunk |
| 717605 | 4751134 | ASS19 | Gould Condensers (GC) | *Robinia pseudoRobinia pseudoacacia* | Bark and internal trunk |
| 717598 | 4751166 | ASS20 a/b | Gould Condensers (GC) | *Verbascum Thapsus* | Bark and external root, foliage |
| 717619 | 4751137 | ASS21 | Gould Condensers (GC) | *Sambucus nigra* | Bark and internal trunk, medulla trunk |

**Table S2** DHA (µg TPF g^-1^day^-1^), total Hg: Hg_tot_ (mg/kg), leached Hg: Hg_l_ (µg/L), soil leachable Hg: Hg_SL_(mg/kg); Organic Hg (%), Inorganic Hg (%), pH; n.d. not determined

| Soil Sample | DHA | Hg_tot_ | Hg_l_ | Hg_SL_ | Organic Hg | Inorganic Hg | pH |
| --- | --- | --- | --- | --- | --- | --- | --- |
|  | µg TPF g^-1^day^-1^ | mg/kg | µg/L | mg/kg | % | % |  |
| ASS1 | 7.5 | 45 | 0.1 | 0.04 | n.d | n.d | 8.48 |
| ASS2 | <1 | 2 | <0.1 | n.d. | 41.6 | 58.4 | 7.98 |
| ASS3 | 11.2 | 158 | 0.3 | 0.12 | 60.36 | 39.64 | 8.55 |
| ASS4 | 68.8 | 1031 | 9.1 | 3.82 | 63.22 | 36.78 | 8.17 |
| ASS5 | 17.6 | 154 | 0.1 | 0.04 | 23.04 | 76.96 | 8.67 |
| ASS6 | 9.7 | 393 | 0.1 | 0.04 | 14.71 | 85.29 | 8.83 |
| ASS7 | 49.6 | 179 | 0.1 | 0.04 | 18.55 | 81.45 | 8.67 |
| ASS8 a | 20.2 | 832 | 0.1 | 0.04 | 6.14 | 93.86 | 8.81 |
| ASS8 b | 166 | 630 | 0.2 | 0.08 | 18.87 | 81.13 | 8.76 |
| ASS9 | 39.4 | 473 | 0.2 | 0.08 | 33.42 | 66.58 | 8.39 |
| ASS10 | 35 | 233 | 0.3 | 0.12 | 59.08 | 40.92 | 8.31 |
| ASS11 | 14.3 | 77 | 0.1 | 0.04 | 34.39 | 65.61 | 8.32 |
| ASS12 | 28.4 | 119 | 0.4 | 0.16 | 49.78 | 50.22 | 8.23 |
| ASS13 | 14 | 512 | 0.3 | 0.12 | 27.18 | 72.82 | 8.83 |
| ASS14 | 11.8 | 517 | 20.4 | 8.56 | 73.53 | 26.47 | 8.52 |
| ASS15 | 32.1 | 493 | 13.4 | 5.62 | 46.07 | 53.93 | 8.56 |
| ASS16 | 68.3 | 67 | 0.2 | 0.08 | 44.4 | 55.6 | 8.48 |
| ASS17a | 38.2 | 772 | 0.2 | 0.08 | 13.74 | 86.26 | 8.61 |
| ASS17b | 151.3 | 604 | 0.3 | 0.12 | 67.39 | 32.61 | 8.68 |
| ASS18 | 113.1 | 560 | 4.9 | 2.05 | 50.85 | 49.15 | 8.4 |
| ASS19 | 70.2 | 782 | 2.3 | 0.96 | 65.86 | 34.14 | 7.93 |
| ASS20 a | 76.1 | 716 | 3.7 | 1.55 | 73.51 | 26.49 | 8.5 |
| ASS20 b | 123.5 | 664 | 1 | 0.42 | 46.03 | 53.97 | 8.42 |
| ASS21 | 122.5 | 1068 | 5.6 | 2.35 | 7.15 | 92.85 | 8.42 |

**Table S3** Bioaccumulation Factor (BF) and mercury content (Hg_plant_ in mg/kg) in different analyzed plants parts. Numbers before the plant species correspond to the soil where they were collected; numbers after the plant species represent different samples from the same plant, and the acronyms TMB (Transport Mercury Belt), GO (Goroncino), FN (Forno Nesa), and CG (Gould Condensers) refer to the four different sites in the mining area from where plants and soils were sampled (see Fig. 1b); n.a. not avaible

| Samples | Hg_plant_ (mg/kg) | BF |
| --- | --- | --- |
| 1 - *Cytisus Scoparius* 1.1 TMB (Bark Root) | 0.97 | 0.023 |
| 1 - *Cytisus Scoparius* 1.2 TMB (Bark Root) | 0.97 | 0.023 |
| 1 - *Cytisus Scoparius* 1.1 TMB (Bark Trunk) | 0.73 | 0.017 |
| 1 - *Cytisus Scoparius* 1.2 TMB (Bark Trunk) | 0.73 | 0.017 |
| 1 - *Cytisus Scoparius* 1.1 TMB (Internal Root) | <0.0005 | n.a. |
| 1 - *Cytisus Scoparius* 1.2 TMB (Internal Root) | <0.0005 | n.a. |
| 1 - *Cytisus Scoparius* 1.1 TMB (Internal Trunk) | 0.02 | <0.001 |
| 1 - *Cytisus Scoparius* 1.2 TMB (Internal Trunk) | 0.02 | <0.001 |
| 1 - *Cytisus Scoparius* 1.1 TMB (External Root) | 2.51 | 0.059 |
| 1 - *Cytisus Scoparius* 1.2 TMB (External Root) | 0.96 | 0.022 |
| 1 - *Cytisus Scoparius* 1.1 TMB (Foliage) | 0.51 | 0.012 |
| 1 - *Cytisus Scoparius* 1.2 TMB (Foliage) | 0.34 | <0.001 |
| 2 - *Cytisus Scoparius* TMB (Bark Root) | 7.43 | n.a. |
| 2 - *Cytisus Scoparius* TMB (Bark Trunk) | 2.61 | n.a. |
| 2 - *Cytisus Scoparius* TMB (Internal Root) | 0.19 | n.a. |
| 2 - *Cytisus Scoparius* TMB (Internal Trunk) | 0.22 | n.a. |
| 2 - *Cytisus Scoparius* TMB (External Root) | 6.84 | n.a. |
| 2 - *Cytisus Scoparius* TMB (Foliage) | 14.36 | n.a. |
| 3 – *Popolus spp.* 1.1 TMB (Bark Root) | 16.86 | 0.13 |
| 3 - *Popolus spp*. 1.2 TMB (Bark Root) | 6.58 | 0.050 |
| 3 - *Popolus spp.*  1.1 TMB (Bark Trunk) | 3.78 | 0.030 |
| 3 - *Popolus spp.* 1.2 TMB (Bark Trunk) | 1.21 | 0.010 |
| 3 - *Popolus spp.* 1.3 TMB (Bark Trunk) | 3.78 | 0.030 |
| 3 - *Popolus spp.* 1.1 TMB (Internal Root) | 0.66 | 0.005 |
| 3 - *Popolus spp*.1.2 TMB (Internal Root) | 0.41 | 0.003 |
| 3 - *Popolus spp.* 1.1 TMB (Internal Trunk) | 1.24 | 0.010 |
| 3 - *Popolus spp* 1.2 TMB (Internal Trunk) | 0.28 | 0.002 |
| 3 - *Popolus spp* 1.3 TMB (Internal Trunk) | 1.24 | 0.010 |
| 3 - *Popolus spp* 1.1 TMB (External Root) | 10.26 | 0.081 |
| 3 – *Popolus spp*.1.2 TMB (External Root) | 2.33 | 0.019 |
| 4 – *Robinia pseudoacacia* TMB (Bark Trunk) | 0.52 | <0.001 |
| 4 - *Robinia pseudoacacia* TMB (Internal Trunk) | 0.68 | <0.001 |
| 5 - *Castanea Sativa* 1.1 TMB (Bark Trunk) | 23.75 | 0.565 |
| 5 - *Castanea Sativa* 1.2 TMB (Bark Trunk) | 22.53 | 0.536 |
| 5 - *Castanea Sativa* 1.3 TMB (Bark Trunk) | 15.21 | 0.362 |
| 5 - *Castanea Sativ*a 1.1 TMB (Internal Trunk) | 3.68 | 0.087 |
| 5 - Castanea Sativa 1.2 TMB (Internal Trunk) | 3.31 | 0.078 |
| 5 - *Castanea Sativa* 1.3 TMB (Internal Trunk) | 3.68 | 0.087 |
| 6 - *Castanea Sativa* TMB (Bark Root) | 7.43 | 0.176 |
| 6 - *Castanea Sativa* TMB (Bark Trunk) | 15.21 | 0.362 |
| 6 - *Castanea Sativa* TMB (Internal Root) | 1.14 | 0.027 |
| 6 - *Castanea Sativa* TMB (Internal Trunk) | 1.11 | 0.026 |
| 6 - *Castanea Sativa* TMB (Foliage) | 7.70 | 0.183 |
| 6 - *Castanea Sativa* TMB (Medulla Root) | 1.21 | 0.028 |
| 6 - *Castanea Sativa* TMB (Medullla Trunk) | 1.08 | 0.025 |
| 7 - *Sambucus* *nigra* 1.1 TMB (Bark Root) | 18.92 | 0.450 |
| 7 - *Sambucus* *nigra* 1.2 TMB (Bark Root) | 39.42 | 0.938 |
| 7 - *Sambucus* *nigra* 1.1 TMB (Bark Trunk) | 26.63 | 0.634 |
| 7 - *Sambucus* *nigra* 1.2 TMB (Bark Trunk) | 26.63 | 0.634 |
| 7 - *Sambucus* *nigra* 1.1 TMB (Internal Root) | 7.09 | 0.168 |
| 7- *Sambucus* *nigra* 1.2 TMB (Internal Root) | 6.65 | 0.158 |
| 7 - *Sambucus* *nigra* 1.1 TMB (Internal Trunk) | 14.05 | 0.334 |
| 7 - *Sambucus* *nigra* 1.2 TMB (Internal Trunk) | 6.15 | 0.146 |
| 7 - *Sambucus* *nigra* TMB (External Root) | 11.28 | 0.268 |
| 7 - *Sambucus* *nigra* TMB (Foliage) | 15.87 | 0.377 |
| 7 - *Sambucus* *nigra* TMB (Medulla Trunk) | 6.26 | 0.149 |
| 7 - *Sambucus* *nigra* TMB (Medulla Trunk) | 3.75 | 0.089 |
| 8 - *Verbascum Thapsus* TMB (Bark Root) | 23.68 | 0.563 |
| 8 - *Verbascum Thapsus* TMB (Internal Root) | 0.04 | 0.001 |
| 8 - *Verbascum Thapsus* TMB (External Root) | 17.68 | 0.420 |
| 8 - *Verbascum Thapsus* TMB (Foliage) | 9.80 | 0.233 |
| 9 - *Robinia pseudoacacia* 1.1 FN (Bark Root) | 3.67 | 0.043 |
| 9 - *Robinia pseudoacacia* 1.2 FN (Bark Root) | 2.47 | 0.029 |
| 9 - *Robinia pseudoacacia* 1.1 FN (Bark Trunk) | 2.87 | 0.034 |
| 9 *- Robinia pseudoacacia* 1.2 FN (Bark Trunk) | 2.87 | 0.034 |
| 9 - *Robinia pseudoacacia* 1.1 FN (Internal Root) | 0.30 | 0.003 |
| 9 - *Robinia pseudoacacia* 1.2 FN (Internal Root) | 0.30 | 0.003 |
| 9 - *Robinia pseudoacacia* 1.1 FN (Internal Trunk) | 0.16 | 0.001 |
| 9 - *Robinia pseudoacacia* 1.2 FN (Internal Trunk) | 0.16 | 0.001 |
| 9 - *Robinia pseudoacacia* 1.1 FN (External Root) | 18.40 | 0.219 |
| 9 - *Robinia pseudoacacia* 1.2 FN (External Root) | 18.40 | 0.219 |
| 10 – *Popolus spp.* GO (Bark Trunk) | 0.56 | 0.003 |
| 10 - *Popolus spp.* GO (Internal Trunk) | 0.34 | 0.018 |
| 11 *– Salix spp.* GO (Bark Root) | 6.17 | 0.146 |
| 11 - *Salix spp* 1.1 GO (Bark Trunk) | 1.46 | 0.034 |
| 11 *– Salix spp* 1.2 GO (Bark Trunk) | 1.46 | 0.034 |
| 11 *– Salix spp* GO (Internal Root) | 0.09 | 0.002 |
| 11 *– Salix spp* 1.1 GO (Internal Trunk) | 0.12 | 0.002 |
| 11 *– Salix spp* 1.2 GO (Internal Trunk) | 0.12 | 0.002 |
| 11 *– Salix spp* GO (External Root) | 0.78 | 0.018 |
| 12 - *Cytisus Scoparius* 1.1 GO (Bark Root) | 1.98 | 0.011 |
| 12 - *Cytisus Scoparius* 1.2 GO (Bark Root) | 1.62 | 0.009 |
| 12 - *Cytisus Scoparius* 1.1 GO (Bark Trunk) | 4.65 | 0.027 |
| 12 - *Cytisus Scoparius* 1.2 GO (Bark Trunk) | 4.65 | 0.027 |
| 12 - *Cytisus Scoparius* 1.1 GO (Internal Root) | 0.45 | 0.002 |
| 12 - *Cytisus Scoparius* 1.2 GO (Internal Root) | 0.45 | 0.002 |
| 12 - *Cytisus Scoparius* 1.1 GO (Internal Trunk) | 0.12 | <0.001 |
| 12 - *Cytisus Scoparius* 1.2 GO (Internal Trunk) | 0.12 | <0.001 |
| 12 - *Cytisus Scoparius* 1.1 GO (External Root) | 3.31 | 0.019 |
| 12 - *Cytisus Scoparius* 1.2 GO (External Root) | 3.31 | 0.019 |
| 12 - *Cytisus Scoparius* 1.1 GO (Foliage) | 6.70 | 0.039 |
| 12 - *Cytisus Scoparius* 1.2 GO (Foliage) | 6.70 | 0.039 |
| 13 - *Robinia pseudoacacia* GO (Bark Root) | 9.25 | 0.073 |
| 13 - *Robinia pseudoacacia* 1.1 GO (Bark Trunk) | 1.01 | 0.007 |
| 13 - *Robinia pseudoacacia* 1.2 GO (Bark Trunk) | 1.01 | 0.007 |
| 13 - *Robinia pseudoacacia* GO (Internal Root) | 0.05 | <0.001 |
| 13 - *Robinia pseudoacacia* 1.1 GO (Internal Trunk) | 0.05 | <0.001 |
| 13 - *Robinia pseudoacacia* 1.2 GO (Internal Trunk) | 0.05 | <0.001 |
| 13 - *Robinia pseudoacacia* GO (External Root) | 3.93 | 0.031 |
| 14 - *Castanea Sativa* GO (Bark Root) | 5.30 | <0.001 |
| 14 - *Castanea Sativa* 1.1 GO (Bark Trunk) | 3.44 | <0.001 |
| 14 - *Castanea Sativa* 1.2 GO (Bark Trunk) | 3.44 | <0.001 |
| 14 - *Castanea Sativa* GO (Internal Root) | 0.10 | <0.001 |
| 14 - *Castanea Sativa* 1.1 GO (Internal Trunk) | 0.07 | <0.001 |
| 14 - *Castanea Sativa* 1.2 GO (Internal Trunk) | 0.07 | <0.001 |
| 14 - *Castanea Sativa* GO (External Root) | 5.01 | <0.001 |
| 14 - *Castanea Sativa* GO (Medulla Trunk) | 0.63 | <0.001 |
| 15 - *Sambucus nigra* GO (Bark Trunk) | 2.05 | <0.001 |
| 15 - *Sambucus nigra* GO (Internal Trunk) | 0.26 | <0.001 |
| 15 - *Sambucus nigra* GO (Medulla Trunk) | 0.44 | <0.001 |
| 16 - *Acer pseudoplatanus* GO (Bark Trunk) | 0.67 | 0.008 |
| 16 - *Acer pseudoplatanus* GO (Internal Trunk) | 0.10 | 0.001 |
| 17 - *Verbascum Thapsus* GO (Bark Root) | 7.11 | 0.084 |
| 17 - *Verbascum Thapsus* GO (Internal Root) | 0.43 | 0.005 |
| 17 - *Verbascum Thapsus* GO (External Root) | 25.52 | 0.303 |
| 17 - *Verbascum Thapsus* GO (Foliage) | 54.55 | 0.649 |
| 18 - *Acer pseudoplatanus* (Bark Trunk) | 3.13 | 0.001 |
| 18 - *Acer pseudoplatanus* CG (Internal Trunk) | 0.38 | <0.001 |
| 19 - *Robinia pseudoacacia* CG (Bark Trunk) | 14.58 | 0.015 |
| 19 - *Robinia pseudoacacia* CG(Internal trunk) | 0.23 | <0.001 |
| 20 - *Verbascum Thapsus* CG (Bark Root) | 37.74 | 0.024 |
| 20 *- Verbascum Thapsus* CG (External Root) | 23.35 | 0.015 |
| 20 - *Verbascum Thapsus* CG (Foliage) | 13.04 | 0.008 |
| 21 – *Sambucus nigra* CG (Bark Trunk) | 8.08 | 0.003 |
| 21 - *Sambucus nigra* CG (Internal Trunk) | 1.26 | <0.001 |
| 21 - *Sambucus* nigra CG (Medulla Trunk) | 0.92 | <0.001 |
